# Genome-Wide Analysis of the *JAZ* Gene Family in Potato and Functional Verification of *StJAZ23* Under Drought Stress

**DOI:** 10.3390/ijms26052360

**Published:** 2025-03-06

**Authors:** Zhuanfang Pu, Tianyuan Qin, Yihao Wang, Xiangdong Wang, Ningfan Shi, Panfeng Yao, Yuhui Liu, Jiangping Bai, Zhenzhen Bi, Chao Sun

**Affiliations:** 1 College of Agronomy/State Key Laboratory of Aridland Crop Science, Gansu Agricultural University, Lanzhou 730070, China; puzf@st.gsau.edu.cn (Z.P.);; 2 Food Crops Research Institute, Xinjiang Academy of Agricultural Sciences, Urumqi 830091, China; qinty@st.gsau.edu.cn

**Keywords:** potato, *JAZ* family, *StJAZ23* functional characterization, drought stress, gene expression

## Abstract

The JASMONATE-ZIM DOMAIN (JAZ) repressors are crucial proteins in the jasmonic acid signaling pathway that play a significant role in plant growth, development and response to abiotic stress (such as drought, heat, salinity, and low temperature). In this study, we identified 26 potato *JAZ* genes and classified the corresponding predicted proteins into five subfamilies. All potato JAZ proteins exhibited the expected conserved TIFY (TIF[F/Y] XG) and JAZ domains. Additionally, we identified several stress-responsive *cis*-regulatory elements, notably ABRE and ARE in the promoters of the *JAZ* gene family. Whole transcriptome and gene family expression analysis identified *StJAZ23* as a key gene responding to drought stress in the root tissues of the Atlantic (Atl) and Qingshu 9 (QS9) potato cultivars. The *StJAZ23* gene was cloned, and subcellular localization analysis suggested that the StJAZ23 protein was mainly localized in the nucleus and cell membrane. This study confirmed that *StJAZ23* plays a role in drought stress by analyzing several *StJAZ23* overexpression (OE-3, OE-5, and OE-6) and RNA interference (RNAi-3, RNAi-6, and RNAi-13) transgenic potato lines. The OE lines displayed significantly increased *StJAZ23* expression compared to wild-type (WT) plants, while RNAi lines exhibited significantly reduced expression. The total root length, root tip count, and root surface area were significantly enhanced in OE lines under drought stress, compared to WT plants, whereas RNAi lines showed significant reductions. *StJAZ23* overexpression also increased the activities of SOD, POD, CAT, and root vigor under drought stress and JA and ABA hormone levels were also significantly increased in roots under drought stress. These results highlight the positive role of the *StJAZ23* gene in enhancing potato resilience to drought stress.

## 1. Introduction

The Jasmonate-ZIM domain (JAZ) proteins are key repressors of the JA signaling pathway, and they play a vital role in regulating the plant response to abiotic stress [1,2]. Studies on *JAZ* gene families have been reported in several species. For example, there are 13 *JAZ* family members in Arabidopsis [3], 25 in rice [4], 16 in corn [5], 17 in wheat [6], and 12 in *Solanum lycopersicum* [7]. The functions of many JAZ proteins have been characterized. For instance, 13 *JAZ* genes were identified in tea (*Camellia sinensis*) that play crucial roles in abiotic stress responses, phytohormone crosstalk, as well as growth and development [1]. In wheat, Wang et al. [8] demonstrated that TaJAZ proteins were highly conserved, with all JAZ proteins clustering into 11 subgroups. The *TaJAZ* genes responded to various abiotic stresses, including high salinity, drought, and cold, as well as to phytohormones. Similarly, *FcJAZ* genes from fig were grouped into five classes, each containing relatively complete TIFY and JAZ domains [9]. Studies in rice showed that 23 *JAZ* transcripts were distributed across the six chromosomes, with conserved domains and different protein characteristics [10]. In soybeans, *GmJAZs* responded to heat, drought, and cold stresses [11]. Arabidopsis overexpressing the apple *MdJAZ2* gene showed reduced JA sensitivity and enhanced tolerance to salt and osmotic stress [12]. In rice, the ectopic activation of *OsJAZ8* led to an improved salt tolerance [13], while *OsJAZ1*-dependent activation of the *OsbHLH148* transcription factor enhanced drought tolerance [14]. These findings underscore the broad role of *JAZ* genes in regulating adaptability to abiotic stress.

Roots play a critical role in anchoring plants to soil, as well as water and nutrient uptake [15]. Studies in rice and Arabidopsis reveal that deep roots and efficient gravity-oriented root development are the primary traits of drought resilience [16,17,18]. *JAZ* genes are important regulators of root development. For example, in tomato, *SlJAZ11* is induced by ABA and salt stress to promote root growth, and *SlJAZ5* is induced by JA to promote root growth [7]. Chen et al. [19] found that the ectopic expression of *EbJAZ* regulates primary root growth via the JA signaling pathway, with *EbJAZ4* acting as a positive regulator of root tip growth [20]. The overexpression of *OsJAZ11* in rice alleviates JA’s inhibitory effects on root growth and promotes primary and elite root elongation [21]. Similarly, the overexpression of *GhJAZ3* in Arabidopsis increased hypocotyl and root length, leaf trichome length, and plant height [22]. In *Caragana korshinskii*, *CwJAZ4*/*9* reduces salt sensitivity, which maintains hairy root growth under salt stress by suppressing salt-mediated jasmonate responses [23]. Significant differences in aboveground tissue and root length have been observed between *OsJAZ1* transgenic rice and WT plants under MeJA and ABA treatments [24]. Collectively, these studies highlight the essential role of *JAZ* genes in root development and abiotic stress responses.

Potato is a typical shallow-rooted tuber crop with weak root penetration, making it particularly sensitive to drought and other stresses throughout its growth period [25]. In previous studies, various methods of counteracting drought in potato cultivation, including compost, plastic mulching with ridge-furrow planting (PM), and screening new potato varieties for drought tolerance have been explored. One study shows that compost combined with a decline in NPK fertilizer may be useful in mitigating drought stress in potato cultivation and decreasing fertilizer usage [26]. Plastic mulching with ridge-furrow planting (PM) increases soil water content. The results show that ridge-furrow planting and vertical rotary subsoiling (PMV) increase potato tuber yield and water use efficiency (WUE) under drought stress [27]. Genetic engineering methods can also be used to transfer drought-related genes to other potato varieties to improve the drought tolerance and agronomic traits of crops, increase yield, and thereby improve the benefits of potato cultivation [28,29]. In the present, various genes have been implicated in promoting root growth under abiotic stresses in potatoes. For example, under salt stress, antisense repression of *StubGAL83* resulted in transgenic lines with stunted and underdeveloped roots compared to wild-type plants [30]. Bi et al. [31] demonstrated that the overexpression of *StCDPK13* increased root length, root number, root volume, and root tip number under drought stress. Additionally, transgenic potato plants overexpressing *miR156* showed a significantly increased number of lateral roots [32]. Finally, the overexpression of *StUBC13* promoted favorable root growth under 200 mM mannitol stress, whereas the wild-type plants failed to root [33].

Although *JAZ* genes appear to play roles in several species in imparting abiotic stress resistance and a more drought-favorable root system architecture, little research has been conducted in potato, which is renowned for having a shallow root system and poor drought tolerance. Therefore, the study carried out a global analysis of the *JAZ* gene family in potatoes and identified 26 *StJAZ* genes using phylogenetic analyses and analyzed *JAZ* promoter cis-acting elements and *JAZ* gene expression patterns. Based on these results, this study focused on undertaking a detailed functional genetic analysis of the *StJAZ23* gene. *StJAZ23* was cloned, and transgenic overexpression (OE) and RNA interference (RNAi) potato lines were obtained. The phenotypes, physiological indicators, gene expression, and hormone contents were analyzed in the OE lines and RNAi lines under drought stress to investigate its function in root growth and drought tolerance. The results highlight a positive role for *StJAZ23* in enhancing potato resilience to drought stress and offer a valuable approach for improving drought tolerance in potato.

## 2. Results

### 2.1. Identification of StJAZ Genes

In this study, 26 members of the *StJAZ* family were identified and named *StJAZ1* to *StJAZ26*. The *StJAZ* genes were unevenly distributed across the 12 potato chromosomes (Figure S1). The protein lengths ranged from 107 (*Soltu.DM.08G007160*) to 816 (*Soltu.DM.01G007210*) amino acids (aa), with molecular weights (MWs) varying from 12.5 kD to 90.6 kD, respectively. The results indicated variability in the gene lengths and MWs within the *StJAZ* family. Additionally, most StJAZ proteins (84.6%) had isoelectric points (pIs) greater than 7, indicating that the majority of gene proteins are basic in nature (Table S1).

### 2.2. Multiple Sequence Alignment and Phylogenetic Analysis

**Figure 1:**
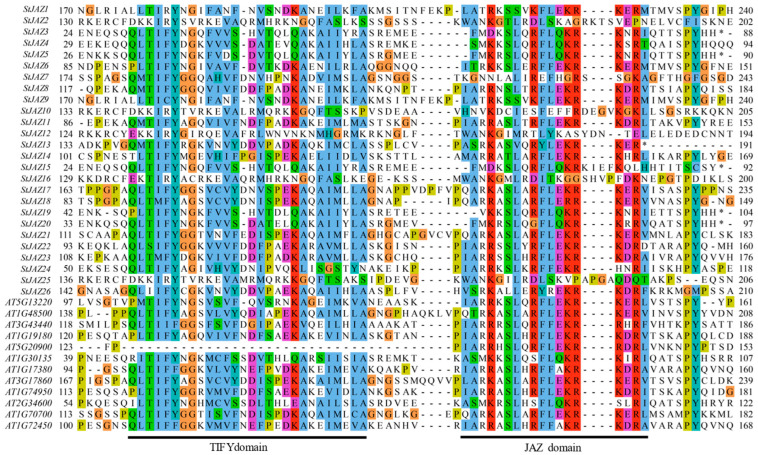
Multiple alignment of the deduced amino acid sequences of *StJAZ* family members. The different colors represent the physico-chemical properties of the amino acids. The blue color represents nonpolar aliphatic amino acids; the green color represents polarized uncharged amino acids; The red color represents positively charged amino acids; the purple color represents negatively charged amino acids; the cyan represents aromatic amino acid; the orange represents hydrophobic nonpolar amino acids; and the yellow color represents imino acids. * represents a fully conserved site (all sequences are identical at this position); and - represents a deletion (Gap), indicates an insertion/deletion mutation (Indel).

**Figure 2:**
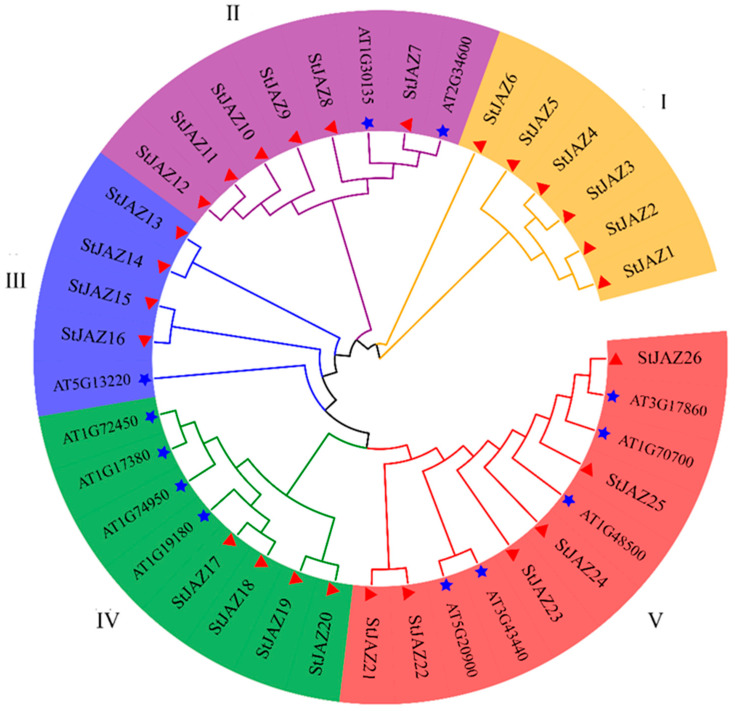
Phylogenetic trees of potato and Arabidopsis JAZ protein sequences. I–V represents the five subfamilies of JAZs. Different colors in the background signify distinct subfamilies. The triangle represents potato and the star represents Arabidopsis.

Alignment of the 26 *StJAZ* amino acid sequences revealed high conservation in the N-terminal TIFY domain, which represents the core region of these gene family members. The JAZ domain, which is associated with responses to abiotic stress, was located at the C-terminus and was present in all family members (Figure 1). To understand evolutionary relationships, a phylogenetic tree was constructed using Arabidopsis and potato JAZ proteins. The results grouped the proteins into five clusters: Groups I, II, III, IV, and V (Figure 2). Group V contained the largest number of members, followed by groups II and IV with equal numbers, while group III contained 5 members.

### 2.3. Promoter Cis-Acting Analysis of StJAZ Genes

**Figure 3:**
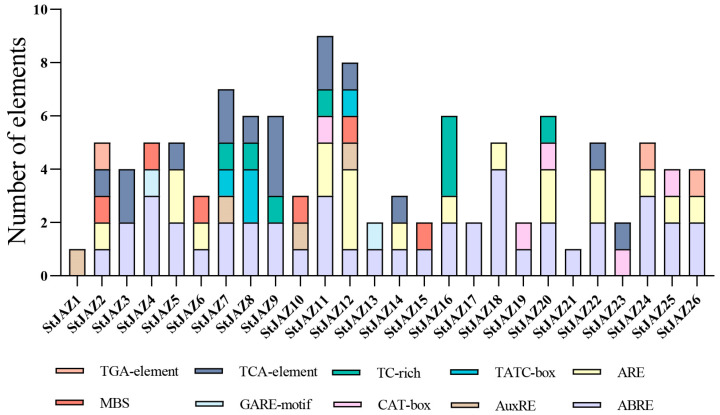
*Cis*-acting elements in *StJAZ* gene promoters. The following elements were identified: abscisic acid response element (ABRE), reactive oxygen species induction-related cis-element (ARE), growth hormone response element (AuxRE), a meristem expression-related (CAT-box), gibberellin response-related element (GARE-motif), drought stress-related action element (MBS), cis-acting element TATC-box involved in gibberellin response, defense and stress response-related acting element (TC-rich), salicylic acid response-related element (TCA-element), and growth hormone response element (TGA-element).

Analysis of *cis*-elements in the promoter regions of the *StJAZ* genes identified 10 functional elements. Prominent elements included drought-inducibility-related (MBS), anaerobic induction-related (ARE), and abscisic acid response (ABRE) motifs. Additionally, a meristem expression-related CAT-box was also detected in the *StJAZ* promoters. These findings suggested that *StJAZs* may have diverse roles in transcriptional regulation and stress responses and that *StJAZs* may respond significantly to ABA and oxidative stress (Figure 3).

### 2.4. Expression Analysis of StJAZ Genes in Response to Abiotic Stress

**Figure 4:**
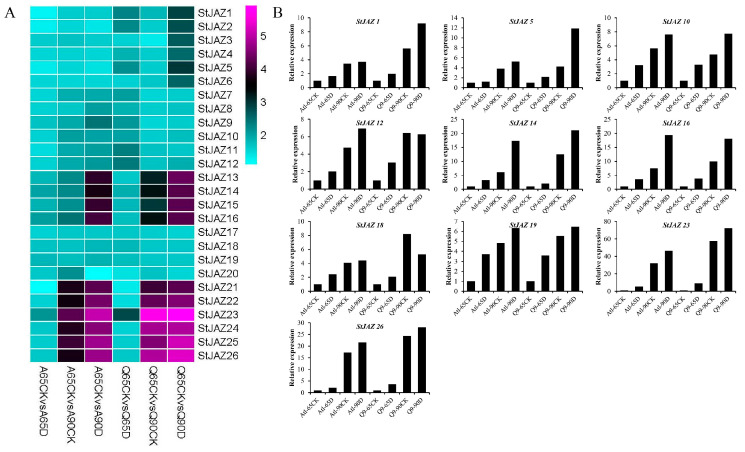
Expression analysis of *StJAZ* genes under different drought stress conditions. (**A**) Expression pattern analysis derived from RNA-Seq data. (**B**) Expression analysis (qRT-PCR).

Transcriptome data were downloaded and analyzed from our previous study [34], whicht revealed varied expression patterns of the 26 *StJAZ* genes under drought stress at 60 and 90 days. Genes in Group I were predominantly expressed at 90 days in the Q9 line, while Group II genes showed high expression in Atl at 90 days. Groups III and V were significantly upregulated over time in both Atl and Q9, whereas Group IV exhibited consistent expression across both cultivars (Figure 4A).

Quantitative qRT-PCR validation of the two most highly expressed genes from each subfamily supported the transcriptome data. Most genes displayed increased expression after 90 days of drought stress, suggesting a positive role for *StJAZ* genes under drought stress. Subsequently, *StJAZ23* was selected for further investigation due to its notable response to drought stress (Figure 4B).

### 2.5. Subcellular Localization of StJAZ23

**Figure 5:**
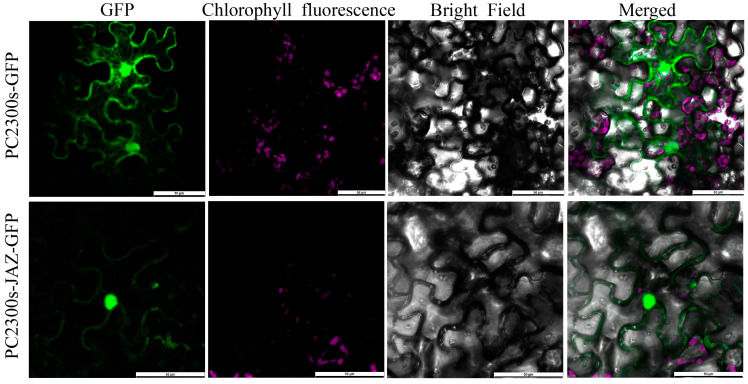
Subcellular localization of the StJAZ23 protein. Bars = 50 μm. Green represents green fluorescent protein and purple represents chloroplast autofluorescence.

To determine the subcellular localization of the StJAZ23 protein, *PC2300s-GFP*, and *PC2300s-JAZ-GFP* fusion protein carriers were transferred into Agrobacterium tumefaciens GV3101 and then injected into tobacco leaves to observe the fluorescence signal. Confocal microscopy revealed that green fluorescence derived from the *PC2300s-JAZ-GFP* was primarily concentrated in the nucleus and cell membrane of tobacco leaf epidermal cells. In contrast, the control *PC2300s-GFP* exhibited fluorescence throughout the cell membrane, cytoplasm, and nucleus (Figure 5).

### 2.6. Generation of Stable Transgenic Potatoes

**Figure 6:**
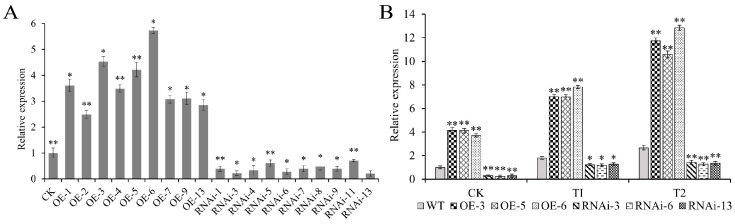
Relative expression of *StJAZ23*. (**A**) Expression level of *StJAZ23* in OE and RNAi lines under control conditions. (**B**) Relative expression of *StJAZ23* under different treatments: CK—control, T1—100 mM mannitol, and T2—200 mM mannitol. Data represent the means ± SD (standard deviation) of three replicates. * and ** indicate significant differences at *p* < 0.05 and *p* < 0.01 levels.

Overexpression (OE) and RNA interference (RNAi) vectors for *StJAZ23* were constructed (Figure S2A,B) and transformed into the E3 potato variety using *A. tumefaciens*. Nine positive OE lines and ten RNAi lines were confirmed using qRT-PCR (Figure S2C and Figure 6A). Three OE (OE-3, OE-5, and OE-6) and RNAi lines (RNAi-3, RNAi-6, and RNAi-13) were selected for further analysis. Expression levels were significantly higher in OE lines and lower in RNAi lines compared to wild-type (WT) plants (Figure 6B). Especially under the T2 condition (200 mM mannitol), the expression levels were increased 3.9–4.8 times in OE lines, while RNAi lines were decreased to 48.2–53.4%.

### 2.7. The Overexpression of StJAZ23 Increases Potato Drought Tolerance

**Figure 7:**
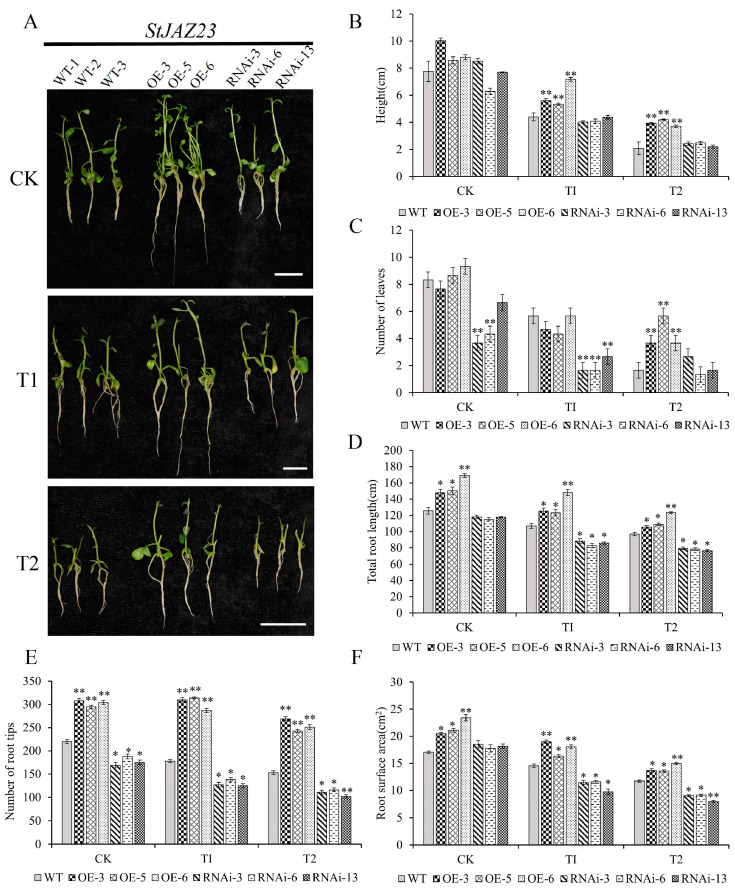
Identification of phenotypes of *StJAZ23* transgenic plants under drought stress. (**A**) Phenotypic assessment of transgenic lines *StJAZ23* and WT plants grown under drought stress for 25 days. CK—control, T1—100 mM mannitol, and T2—200 mM mannitol; (**B**) Plant height; (**C**) Number of leaves; (**D**) Total root length; (**E**) Number of root tips; (**F**) Root surface area. Data represent the means ± SD (standard deviation) of three replicates. * and ** indicate significant differences at *p* < 0.05 and *p* < 0.01 levels. Bars = 20 mm.

To determine the drought tolerance function of *StJAZ23*, transgenic potato and WT plants were subjected to drought stress. Under drought stress, OE lines demonstrated superior phenotypic and growth traits compared to WT plants, while RNAi lines showed inhibited growth (Figure 7A). Parameters such as root length, number of root tips, and root surface area were significantly higher in OE lines as the stress conditions were elevated whereas the root growth of RNAi lines exhibited a more pronounced inhibitory effect (Figure 7D–F). Under T2 mannitol stress, the number of leaves and plant height were significantly higher in OE lines than in WT plants, and RNAi lines showed no significant difference compared with WT plants (Figure 7B,C). These findings suggest that *StJAZ23* positively influences potato growth and development under drought stress.

### 2.8. Overexpression of StJAZ23 Enhances Antioxidant Enzyme Activities and Root Vigor

**Figure 8:**
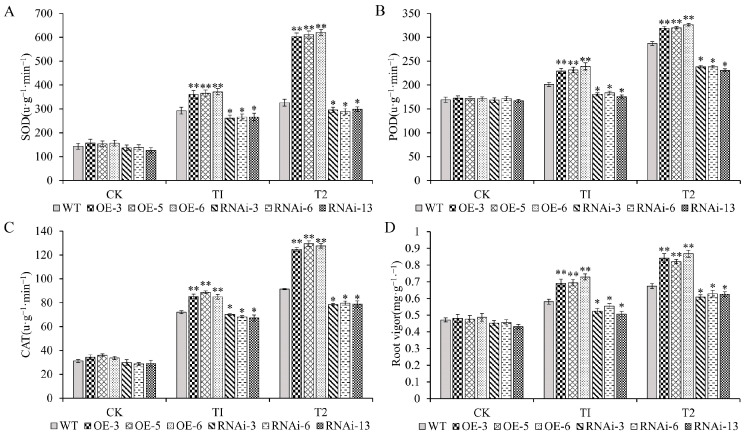
Measurement of antioxidant enzyme activities and root vigor of *StJAZ23* transgenic lines under increasing drought stress. CK—control, T1—100 mM mannitol, and T2—200 mM mannitol; (**A**) SOD activity; (**B**) POD activity; (**C**) CAT activity; (**D**) Root vigor of transgenic potato plants. Data represent the means ± SD (standard deviation) of three replicates. * and ** indicate significant differences at *p* < 0.05 and *p* < 0.01 levels.

The activities of antioxidant enzymes, including POD (peroxidase), SOD (superoxide dismutase), and CAT (catalase), were measured (Figure 8A–C). Under normal conditions, no difference was observed among the OE, RNAi, or WT lines. Under drought stress, however, the activities of POD, SOD, and CAT were significantly increased in OE lines compared to WT plants, whereas RNAi lines exhibited significantly decreased enzyme activities. Notably, these differences became more pronounced with increasing stress intensity. Root vigor, a key physiological indicator closely correlated with drought stress tolerance, was also assessed (Figure 8D). Under normal conditions, root vigor showed no significant differences among the transgenic lines and WT plants. The root vigor of the OE lines, however, was significantly higher than that of the WT plants, whereas the RNAi lines showed significantly lower root vigor under drought stress. Collectively, these results indicate that *StJAZ23* overexpression enhanced the activities of SOD, POD, CAT, and increased root vigor, potentially mitigating oxidative damage during drought stress.

### 2.9. Increased Hormone Content in StJAZ23 Overexpression Lines

**Figure 9:**
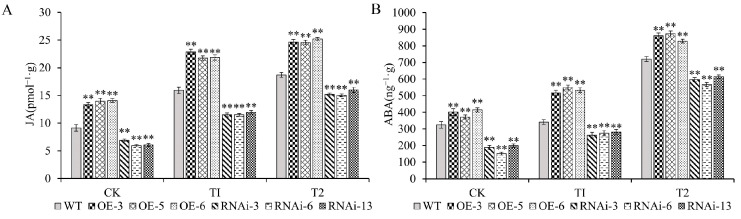
Determination of hormone levels in *StJAZ23* transgenic lines under increasing drought stress. CK—control, T1—100 mM mannitol, and T2—200 mM mannitol; (**A**) JA levels in transgenic potato plants; (**B**) ABA levels in transgenic potato plants. Data represent the means ± SD (standard deviation) of three replicates. ** indicate significant differences at *p* < 0.01 levels.

The levels of two hormones associated with stress tolerance were measured under drought stress (Figure 9A,B). The levels of JA and ABA were significantly higher in the OE lines compared to WT plants, whereas the RNAi lines displayed significantly lower hormone levels. The contents of JA and ABA were especially increased 1.4–1.5 time and 1.5–1.6 times in OE lines, while RNAi lines were decreased to 72.1–75.0% and 76.9–82.4% under the T1 condition. Meanwhile, the contents of JA and ABA were increased 1.3–1.4 times and 1.1–1.2 times in OE lines, while RNAi lines were decreased to 80.3–85.6% and 78.7–85.3% under the T2 condition. These results suggested that *StJAZ23* expression influenced the accumulation of stress-related hormones JA and ABA, contributing to improved drought tolerance.

## 3. Discussion

Potatoes contribute significantly to global food security and socio-economic development [35]. Drought stress, however, negatively impacts potato growth and yield. Genetic engineering provides a promising approach to improve crop resilience, quality, and productivity [36]. *JAZ* plays an important role under abiotic stresses. The *JAZ* gene family is known to play vital roles in plant responses to abiotic stress in other species [37,38,39,40,41]. In tomato [42], all members of the *JAZ* family contain the TIFY and JAZ domains. In this study, 26 *StJAZ* genes were identified in the potato genome and designated *StJAZ1–StJAZ26* (Figure 1). These genes exhibit conserved TIFY and JAZ domains, consistent with findings in tomato. Phylogenetic analysis categorized *StJAZ*s into five subfamilies, with structural differences among subfamilies contributing to their functional diversity. Promoter analysis revealed that most *StJAZ* genes contain ABRE and ARE cis-acting elements (Figure 3), which are associated with ABA responses and oxidative stress, respectively [43].

Drought stress disrupts photosynthesis, restricts root growth, and alters root architecture due to thicker and shorter root branches [44,45]. Several studies have demonstrated that genetic modifications can improve root traits under abiotic stress. It was shown that the heterologous expression of *ZmNF-YA12* in Arabidopsis resulted in increased root length and better plant growth than in WT plants under conditions of mannitol, salt, and JA stress on 1/2 MS medium [46]. The absence of *AtRHD6* impaired root hair development while the overexpression of *RHD6* promoted the development of root hair by regulating JA synthetic signaling [47]. Similarly, under drought stress, the overexpression of *StJAZ23* promoted root development, increased root length, surface area, and number of root tips, whereas RNAi lines showed reduced root growth. These results suggest that *StJAZ23* positively regulates root development during drought stress. This gene can be transferred to other potato varieties to improve the agronomic traits of crops, increase root development and drought resistance using genetic engineering methods, providing methods for obtaining new potato strains of deep-rooted drought tolerance. [28].

Oxidative stress induced by drought leads to excessive production of ROS, necessitating enhanced antioxidant enzyme activities for mitigation [48,49]. Prior studies have shown increased SOD, POD, and CAT activities in genetically modified plants under abiotic stress. Xin et al. [50] reported that POD, CAT, and SOD activities were increased in *Sophora viciifolia* seedlings under drought stress, enhancing ROS scavenging and revealing a heightened response to oxidative stress. Similarly, Jin et al. [51] found in cotton that under NaCl and PEG stress, RNAi plants of *GhANN4* showed more pronounced leaf wilting and reduced CAT, POD, and SOD activities. Furthermore, overexpression of *VvNAC08* in Arabidopsis lines increased the activities of CAT, SOD, and POD under drought stress [52]. Another study showed that SOD, POD, CAT, and APX activities and gene expression were higher in tobacco expressing the *BcICE1* gene compared to those of wild type under cold stress [53]. Root vigor is an important indicator of plant growth and health. Studies have shown that overexpression of *TrSOS1* enhanced salt tolerance by maintaining higher root vigor in cotton [54]. The GmTIR1A-RNAi plants displayed lower root vigor compared to empty-vector plants, indicating that knocking down *GmTIR1A* decreases water stress tolerance [55]. In this study, the *StJAZ23* OE lines exhibited significantly higher antioxidant enzyme activities and root vigor under drought stress, whereas the RNAi lines showed reduced antioxidant enzyme activities. The increased antioxidant enzyme activities in the *StJAZ23* OE lines would potentially mitigate peroxide damage.

JA and ABA are key hormones regulating plant responses to abiotic stress [56]. Studies have reported that drought stress exerts a series of effects on the synthesis, signaling, and transport of plant hormones, playing a crucial role in drought stress response and adaptation [57]. For example, overexpression of the *AtTPPI* gene increased JA content in transgenic plants, enhancing cold tolerance under low-temperature treatment [58]. Similarly, plants overexpressing *IbGATA24* showed significantly increased ABA and JA levels, which enhanced tolerance to drought and salt stress [59]. The study showed that JA content and the expression of JA signaling–related genes decreased in the *SmLOX4*- and *SmLOX5*-silenced plants but increased in the OE-*SmLOX4* and OE-*SmLOX5* transgenic plants [60]. Xu et al. [61] found that overexpression of *SlMYC2* promoted the accumulation of JA and ABA in leaves under drought stress. Overexpression of *CkPYL4* was shown to enhance endogenous ABA accumulation in roots, significantly improving salt and drought tolerance in *Arabidopsis thaliana* and *84 K* poplar plants [56]. Zhang et al. [62] identified *BcSRC2*, a gene containing a C2 domain that responds to ABA signaling and drought regulation in Pak choi. *BcSRC2* interacts with *BcAPX4,* increasing the ABA content under drought stress. In this study, the overexpression of *StJAZ23* increased JA and ABA levels, which correlated with enhanced drought tolerance. These results align with previous findings, such as the interaction of *ZmHsf28* with *ZmSnRK2.2* and *ZmJAZ14/17*, which elevated ABA and JA accumulation to confer drought tolerance in maize [63].

## 4. Materials and Methods

### 4.1. Identification of Potato StJAZ Genes

The *StJAZ* gene family in the potato genome was identified using the latest reference genome sequence from the Spud DB database (http://spuddb.uga.edu/dm_v6_1_download.shtml (accessed on 23 February 2023)). Multiple searches were conducted using the Hidden Markov Model (HMM) of gene families (*JAZ* registry numbers: PF09425, PF06200) and local BLAST. Additionally, Arabidopsis *JAZ* gene sequences (*AtJAZ*) were retrieved from the plant whole genome database Phytozome (https://phytozome-next.jgi.doe.gov/ (accessed on 25 February 2023)) as seed sequences. The BLAST 2.2.25 was used to identify *StJAZ* gene family members in potato based on the e-values ≤ 1 × 10^−20^. The protein sequences of the *StJAZ* family members were aligned and submitted to the CDD (https://www.ncbi.nlm.nih.gov/Structure/cdd/cdd.shtml (accessed on 10 March 2023)) and Pfam databases (http://pfam.xfam.org/ (accessed on 18 February 2024)), respectively, for confirmation, ensuring that identified genes matched the conserved *StJAZ* domains.

### 4.2. Phylogenetic Analysis

The identified *StJAZ* genes underwent phylogenetic analysis using the CDD database (https://www.ncbi.nlm.nih.gov/Structure/cdd/wrpsb.cgi (accessed on 10 March 2023)). Protein sequences were aligned using ClustalX (version 1.83) [64] with default system parameters. A MEGA file was generated for constructing a phylogenetic tree in MEGA7 software [65] using the maximum likelihood method with a bootstrap value of 1000. Based on the phylogenetic tree topology, potato *StJAZ* gene members were categorized into subfamilies. The ExPASy database (http://web.expasy.org/protparam/ (accessed on 25 March 2024)) was used to compute amino acid counts, molecular weights, and isoelectric points (pI) for *StJAZ* gene members.

### 4.3. Analysis of StJAZs Gene Promoter Cis-Acting Element

Using Perl 5 software, sequences 1.5 kb upstream of the coding region of the potato *StJAZ* genes were extracted and submitted to the PlantCARE database (http://bioinformatics.psb.ugent.be/webtools/plantcare/html/ (accessed on 8 April 2024)) to identify key *cis*-acting elements.

### 4.4. Expression Pattern Analysis of Potato StJAZ Gene Family Members

The transcriptome data of *StJAZ* genes were selected from our previous study [34], which were downloaded from the National Center for Biotechnology Information (NCBI) BioProject database under accession number PRJNA1032423 and iProX database under accession number PXD046520, involving potted plantings of “Atlantic” (Atl) and “Qingshu 9” (QS9) under normal watering and severe drought stress conditions at 60 and 90 days, respectively. To further investigate the expression pattern of different potato *StJAZ* genes, the Heatmap package in R 4.2.1 software was used to create a heatmap of gene expression patterns. The expression levels of key genes were analyzed using qRT-PCR in combination with RNA-seq data.

### 4.5. Plant Material and Growth Conditions

Potato cultivars “E3” (E potato No. 3, wild type, WT, derived from Southern China Potato Center), tobacco (*Nicotiana benthamiana*), and transgenic potatoes overexpressing and exhibiting RNA interference (RNAi) of *StJAZ23* were used in this study. The potato seedlings were micropropagated in culture bottles on a modified MS medium with or without mannitol, representing drought stress and control conditions, respectively. *StJAZ23*-overexpression (OE) lines, RNA interference (RNAi) lines, and WT (E3) potato plants were cultured on MS medium under simulated drought conditions: 0 mM mannitol as the control (CK), 100 mM mannitol as moderate drought (T1), and 200 mM mannitol as severe drought (T2). For the simulated drought experiment, five seedlings were grown in each culture bottle, with fifteen replicate bottles examined for each line and treatment. Potato plants were grown in an artificial growth chamber with a 16-h light and 8-h dark cycle, at 22 ± 2 °C, and 60% relative humidity. Tobacco seedlings were grown in a greenhouse under a 12-h light/dark cycle at 25 ± 2 °C and a 60% relative humidity.

### 4.6. Cloning and Generation of Transgenic Potato Plants of StJAZ23

Total RNA was extracted from potato plants, and first-strand cDNA synthesis was performed using the kit protocol (TOYOBO, Osaka, Japan). The coding sequence (CDS) of the *StJAZ23* gene was amplified by PCR using gene-specific primers. The PCR product was subsequently digested with *SacI* and *BamHI* restriction enzymes and ligated into the *PC2300s-GFP* vector to generate the recombinant overexpression plasmid *PC2300s-StJAZ23-GFP*. For RNA interference (RNAi), forward fragments were digested with *HindIII* and *XbaI* and ligated into the *CAM-RNAi* vector to create the RNAi plasmid (*CAM-RNAi-StJAZ23*). Both constructs were transformed into *Agrobacterium tumefaciens* GV3101 cells using the heat-shock method, and subsequently introduced into potato plants (E3) using the A. tumefacien-mediated method according to the procedure of Grandellis et al. [66]. In addition, positive lines were identified using specific primers by PCR. Finally, the expression levels of *StJAZ23* were detected in each positive line using qRT-PCR.

### 4.7. Subcellular Localization of StJAZ in Tobacco

To investigate the subcellular localization of the StJAZ23 protein, the vectors *PC2300s-GFP* and *PC2300s-StJAZ23-GFP* fusion protein carriers were transferred into *A. tumefaciens* GV3101. Then, *A. tumefaciens* GV3101, which contained plasmids carrying the target gene, were incubated overnight in 4 mL LB liquid culture medium (4 µL Rif, 4 µL Kan, 10 mM MES, and 20 µM AS) at 28 °C and oscillated at 180 rpm. The bacteria were collected by centrifugation (5000 rpm, 5 min), resuspended to OD600 = 0.4, transferred into tobacco leaves by the injection method, and cultured at 25 °C for 48 h in darkness. GFP fluorescence signals were visualized with a laser scanning confocal microscope (Leica, Weztlar, Germany).

### 4.8. Gene Expression Analysis Under Drought Stress

RNA was extracted using a total plant RNA extraction kit (Tiangen Biotech, Beijing, China). RNA purity and concentration were measured using an ultra-micro spectrophotometer (Roche, Basel, Switzerland), and then reverse transcribed to cDNA using the kit protocol (TOYOBO, Osaka, Japan), and stored at −20 °C for subsequent use. Fluorescent primers for *StJAZ23* were designed, and gene expression was assessed via qRT-PCR using actin as the internal reference (Table S2). Data were collated and analyzed by ANOVA using Microsoft Excel 2021 and SPSS 22.0 software. The relative expression of the *StJAZ23* gene was calculated using the 2^−ΔΔCT^ method.

### 4.9. Phenotypic, Physiological and Hormonal Analysis of Potato Plants Under Drought Stress

*StJAZ23*-overexpression, RNA interference, and WT potato plants were cultured on MS medium with 0 mM, 100 mM, and 200 mM mannitol for 25 days, before the phenotypic and physiological indicators were determined. The number of leaves was recorded for each plant in all the treatments. The plant height was measured with a meter rule at the time of sampling. Several root indices, including the root length, root surface area, and number of root tips, were measured on both stressed and unstressed plants using a Root Scanner (STD) 4800 (EPSON, Quebec City, QC, Canada). Root physiological indicators were determined using several kits that measured enzymatic activity or hormone levels in circa 200 mg of root tissue that was ground in liquid nitrogen prior to analysis. The following kits were used: root vigor (NAD(P)^+^ dehydrogenase) activity (ml095012, Mlbio, Shanghai, China), superoxide dismutase (SOD) (ml095266, Mlbio, Shanghai, China), peroxidase (POD) (ml095259, Mlbio, Shanghai, China), and catalase (CAT) (ml095270, Mlbio, Shanghai, China) were used to measure root vigor, SOD, POD, and CAT activities, and jasmonic acid (JA) (ml077234, Mlbio, Shanghai, China) and abscisic acid (ABA) (ml077235, Mlbio, Shanghai, China) were used to measure JA and ABA contents.

### 4.10. Statistical Analysis

All experiments were conducted in triplicate, with three technical repeats. Means and standard deviations were calculated, and one-way ANOVA was performed using SPSS v24.0 (Chicago, IL, USA), for statistical evaluations with the general linear model (GLM). All data are presented as the mean ± S.D. (standard deviation). Post hoc comparisons of the means were performed using Duncan’s Multiple Range Test (DMRT) to assess variability between overexpression (OE) lines, RNAi lines, and wild-type plants. Statistical significance was defined as *p* < 0.05 (*) and *p* < 0.01 (**).

## 5. Conclusions

This study identified 26 *StJAZ* genes in the potato genome, which share conserved structural domains and exhibit functional diversity across subfamilies. Among these, *StJAZ23* was significantly upregulated under drought conditions. The study cloned the potato St*JAZ23* gene and developed overexpression and RNAi transgenic lines. The overexpression of *StJAZ23* in potato enhanced root development, antioxidant enzyme activities, and the accumulation of JA and ABA, thereby improving drought tolerance. *StJAZ23* positively regulates root development and enhances drought tolerance in potato. The findings enrich the molecular regulatory network of potato drought resistance and offer a valuable approach for improving drought tolerance in potato by genetic engineering.

## Data Availability

Data are contained within the article and Supplementary Materials.

## Supplementary Materials

The following supporting information can be downloaded at: https://www.mdpi.com/article/10.3390/ijms26052360/s1.
