# Supplementary material for: Genome-Wide Analysis of the JAZ Gene Family in Potato and Functional Verification of StJAZ23 Under Drought Stress
Source: Int J Mol Sci. 2025 Mar 6;26(5):2360. doi: 10.3390/ijms26052360 (PMC11899781; doi:10.3390/ijms26052360)
Supplement: Supplementary file 1 [file ijms-26-02360-s001.zip › Figure S1-S2.pdf]

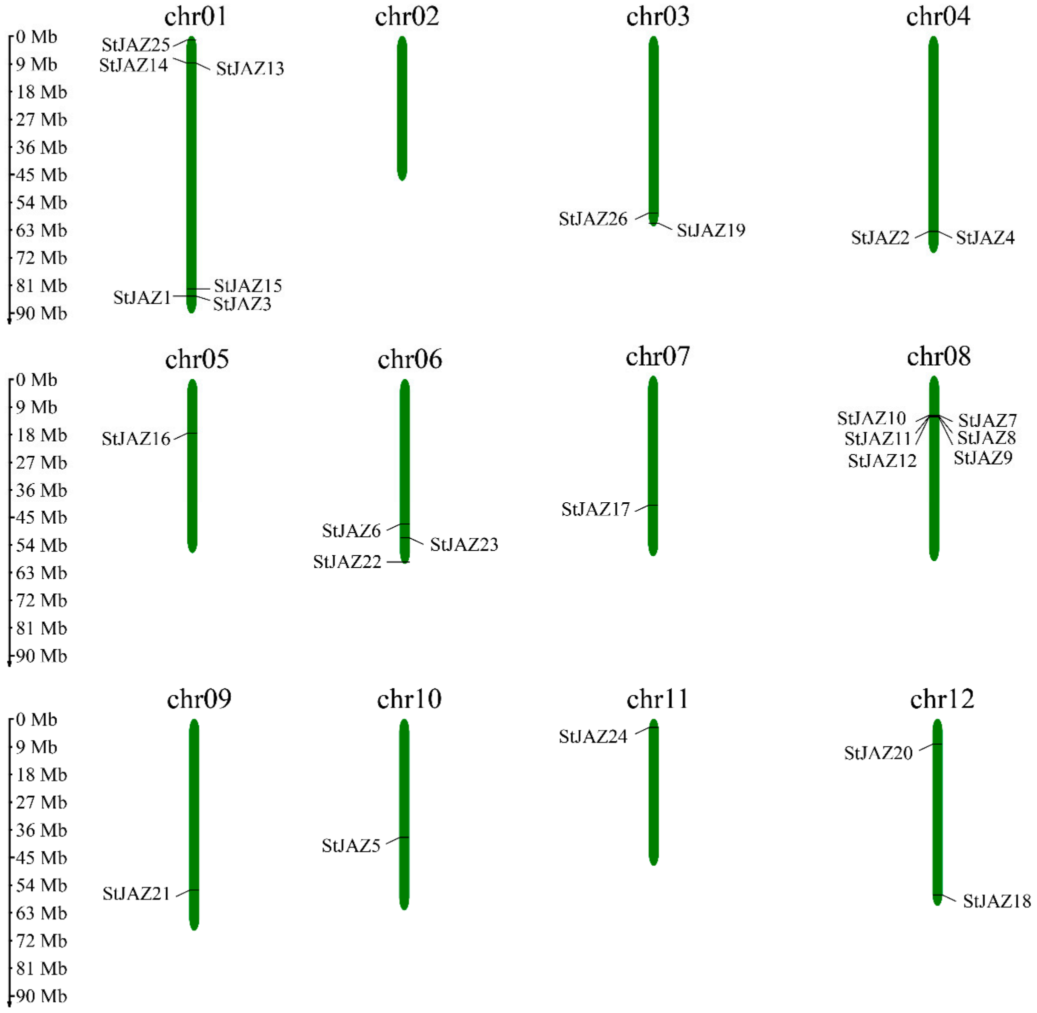

Figure S1. Genomic distributions of *StJAZ* genes on the potato chromosomes

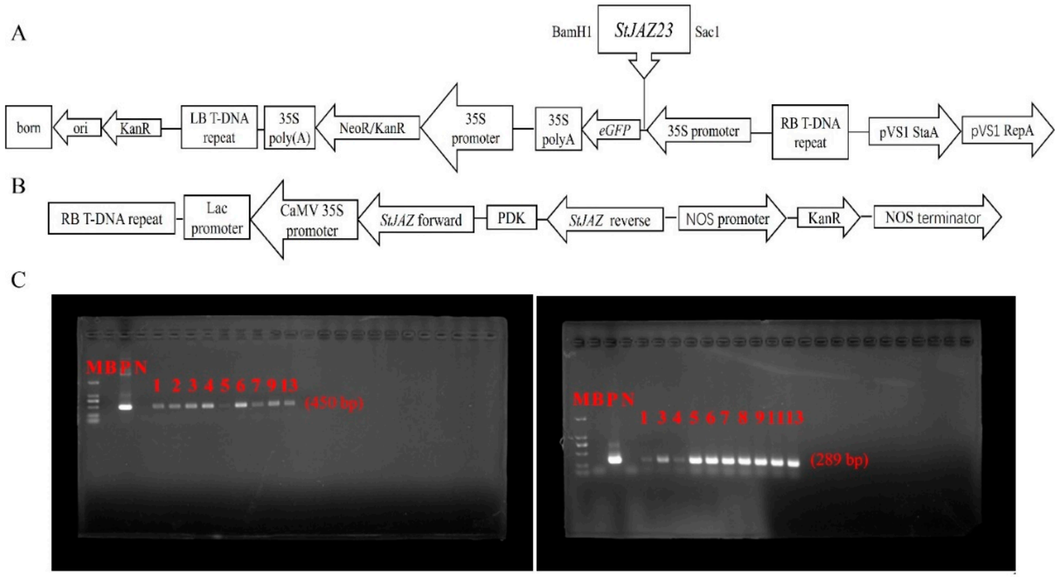

Figure S2. Plasmid map and identification of *StJAZ23*. (A) Schematic representation of the construction of the *StJAZ3* overexpression vector; (B) Schematic representation of the construction of the *StJAZ23* RNAi vector. (C) PCR molecular identification of *StJAZ23* transgenic lines.
